# A Predictable Aesthetic Rehabilitation of Deciduous Anterior Teeth in Early Childhood Caries

**DOI:** 10.1155/2018/1742529

**Published:** 2018-04-03

**Authors:** Priyanka Agarwal, Rashmi Nayak, Ghayathri Elangovan

**Affiliations:** Department of Pedodontics and Preventive Dentistry, Manipal College of Dental Sciences, Manipal 576104, Karnataka, India

## Abstract

Aesthetic dentistry plays a significant role not only in adults but also in pediatric patients. However, a pediatric dentist is faced with the dual problem of satisfying the aesthetic expectations of the patient and parents as well as managing the pediatric patient. In the present era, there are numerous restorative techniques that can be applied to different clinical scenarios. However, we have to choose the technique that best suits our patient, not only biologically but also aesthetically, psychologically, functionally, and financially. The following paper presents the clinical sequence of rehabilitation of severely carious maxillary anterior teeth from left to right lateral incisors in a child with early childhood caries. Severely carious anterior teeth were endodontically treated. The central incisors were restored with gamma loop posts which is mainly used for pediatric patients in endodontically treated teeth. Lateral incisors were treated with Ribbond polyethylene fibre posts. Following this, all the teeth were restored aesthetically with free-hand composite buildup after proper shade selection. The occlusion was restored, and the restorations were finished and polished.

## 1. Introduction

Early childhood caries is an infectious disease of the primary teeth in children which if not intervened at an early stage can lead to severe destruction of the teeth not only in the primary dentition but can also affect their successors [1]. Inspite of the increasing awareness among parents about dental caries and its ill effects, we are frequently faced with situations when we need to extract the teeth with its imminent consequences. The concept of parents insisting for extraction of an extensively decayed tooth of their child has become obsolete. There has been a paradigm shift in the attitude of parents wherein a good portion of the society is more determined to maintain the primary teeth in the oral cavity of their children for as long as they should naturally last. This expectation of the parents cannot be denied, and the final outcome is that more teeth are being restored than used to be during the last century. Not surprisingly, there are diverse techniques and materials [2] that are being used to maintain the primary teeth in the oral cavity of children in a healthy condition. It is the responsibility of the pediatric dentist to choose the technique and the material that best suit the patient's condition. Pediatric dentists have to face the dual challenge of restoring severely decayed teeth at the same time managing the behaviour of the child because children are among the youngest and the least adaptable groups of patients. In addition to management problems, there are a number of procedural problems that need to be addressed while restoring primary incisors. Their crowns are short and narrow, while the pulp chamber is large with respect to the size of the crown [3]. In pulpectomised primary anterior teeth where the entire crown is destroyed by the carious process, only a small amount of the tooth structure is available for bonding. Enamel, if and when present, is also less amenable to acid etching than the permanent teeth because of more aprismatic enamel [4]. In many cases, the entire coronal structure is destroyed, sparing only the root and hence only dentine to bond to the restorative materials. Not surprisingly, in the past, and often even now, many of these teeth are extracted [5]. The following paper elaborates the clinical sequence of rehabilitation of severely mutilated maxillary anterior teeth from left to right lateral incisors in a child with early childhood caries.

## 2. Case Report

**Figure 1:**
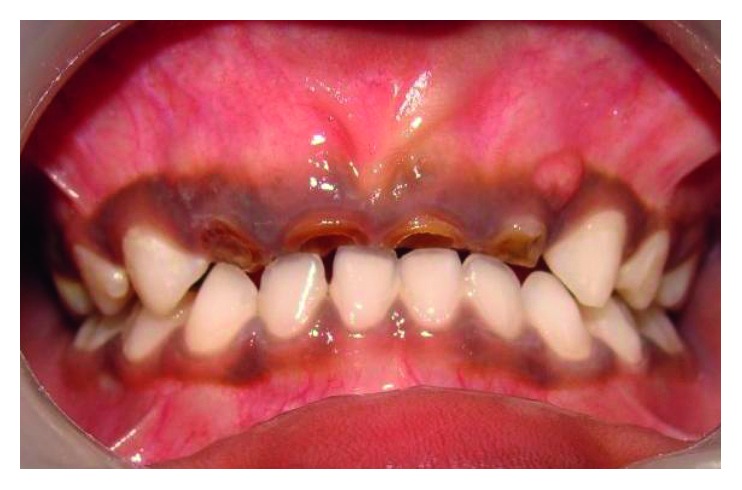
Preoperative frontal view.

**Figure 2:**
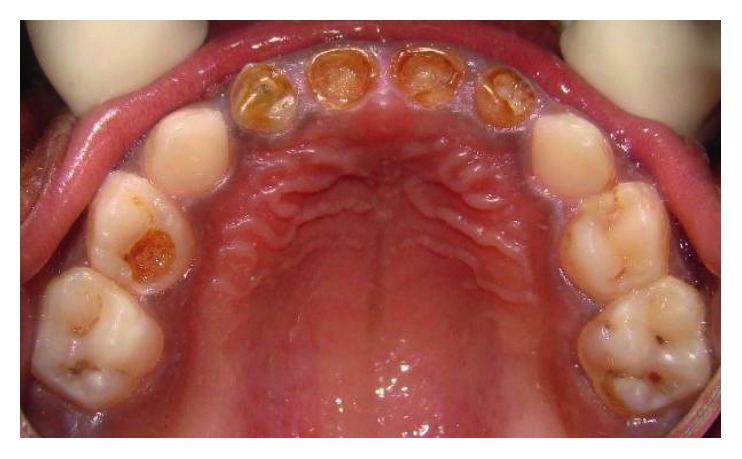
Preoperative maxillary occlusal view.

**Figure 3:**
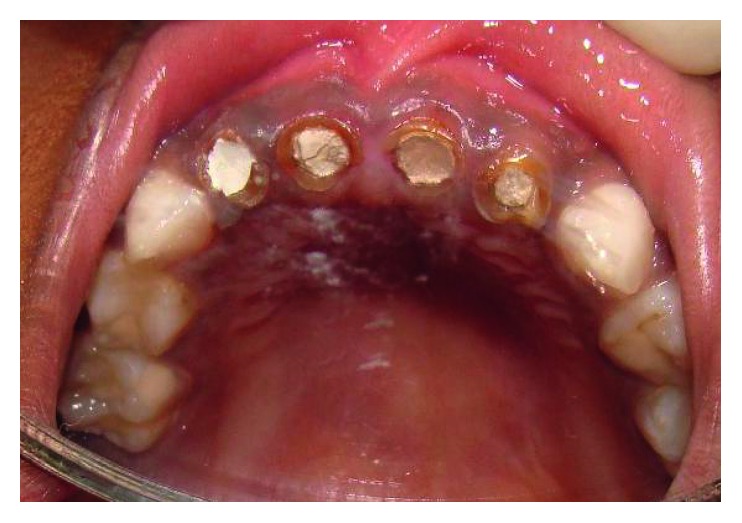
Obturated 51, 52, 61, and 62.

A four-and-a-half-year-old boy reported along with his parents to the Department of Pedodontics and Preventive Dentistry with the chief complaint of decayed teeth in the upper front region since many months. His medical history was not significant. On intraoral examination, he was found to have several carious lesions with grossly decayed 52, 51, 61, and 62 (Figures 1 and 2) and deep dental caries in 85. However, the radiographs of the maxillary anterior teeth revealed good root length of these teeth (Figure 3). Hence, it was planned to restore the maxillary anterior teeth by performing pulpectomy followed by the post and core for these teeth. The parents were informed, and a written consent was obtained.

## 3. Clinical Procedure

**Figure 4:**
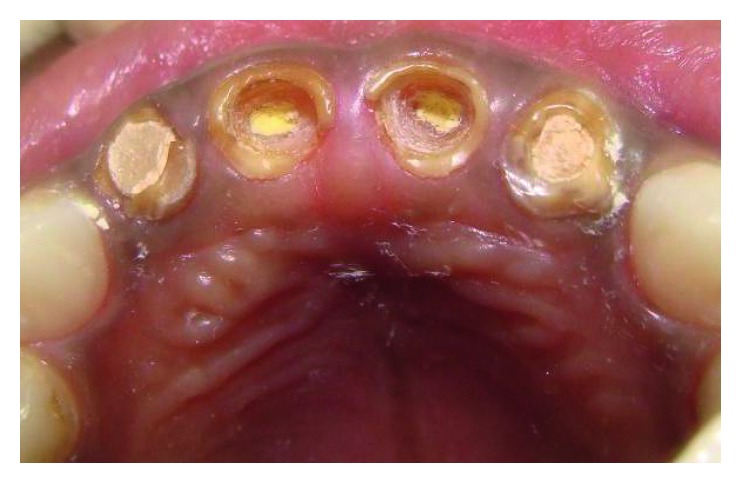
Post space preparation 51 and 61.

**Figure 5:**
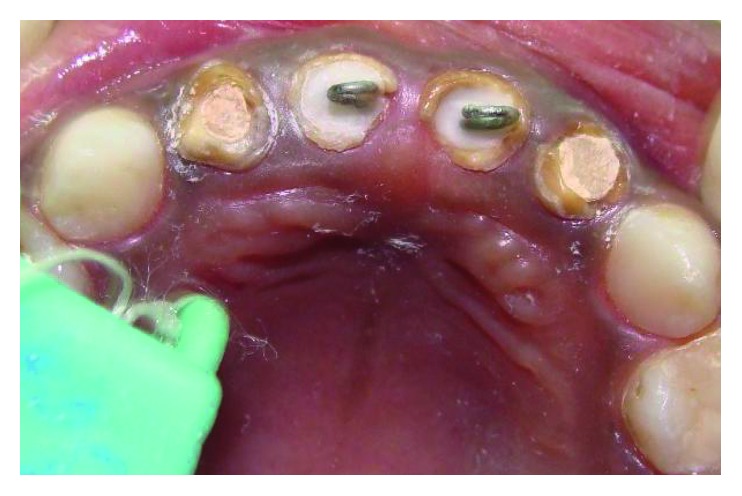
Gamma post cemented.

**Figure 6:**
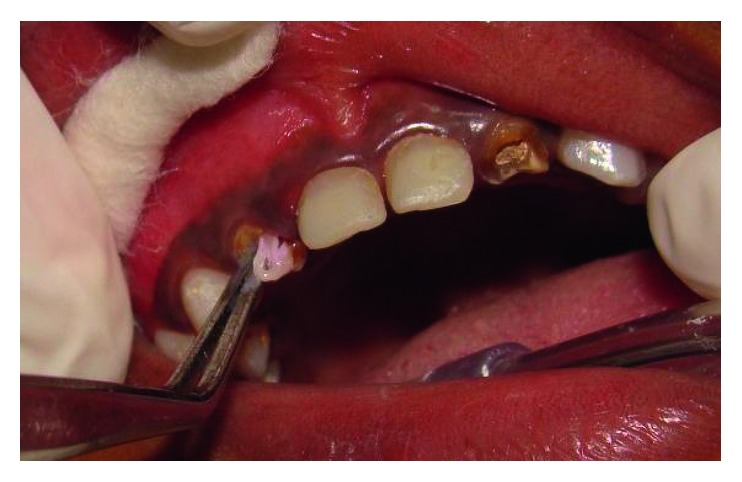
Placement of the Ribbond post.

**Figure 7:**
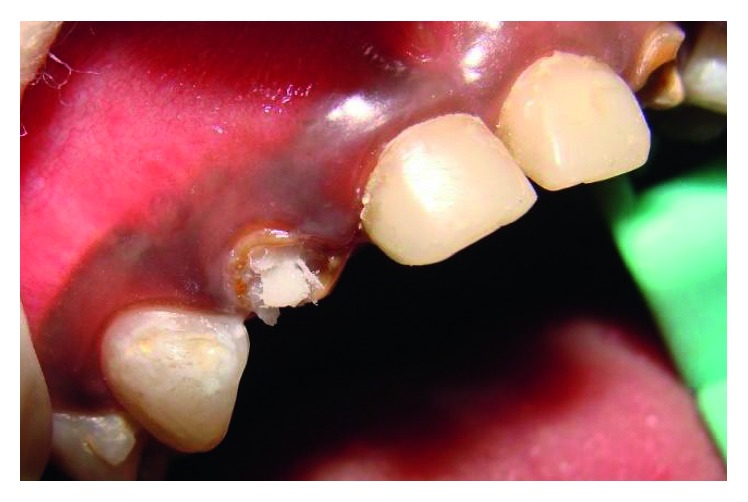
Ribbond post cured.

**Figure 8:**
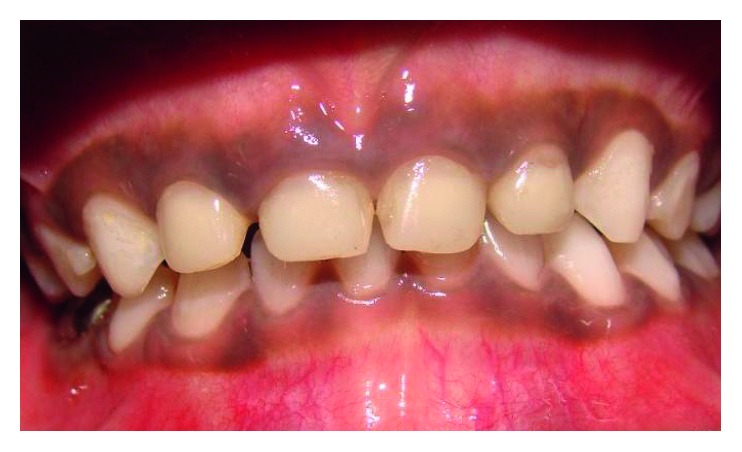
Postoperative frontal view.

**Figure 9:**
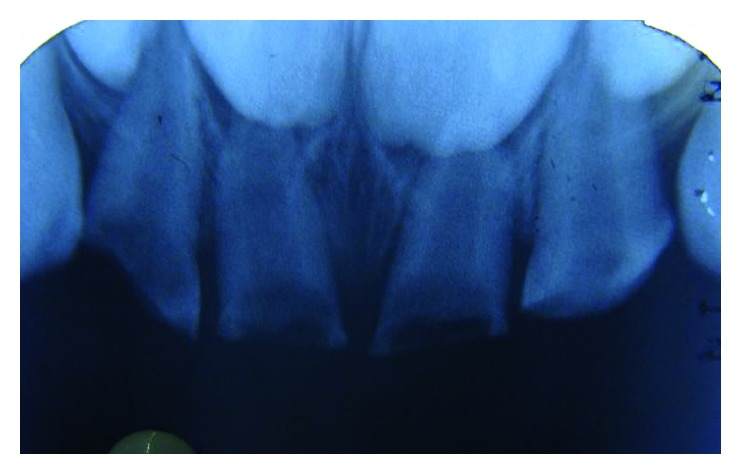
Preoperative IOPAR 52, 51, 61, and 62.

**Figure 10:**
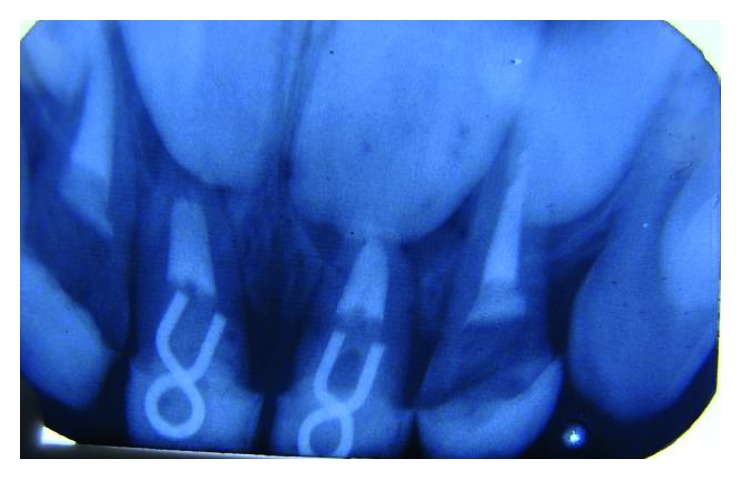
IOPA of 51, 61 showing intraradicular gamma post.

- The maxillary central incisors and lateral incisors were pulpectomised and obturated with Vitapex (Neo Dental Chemical Products Co. Ltd.) (Figure 4).
- Space was created for the intracanal post by removing Vitapex from the canals (coronal 3-4 mm) (Figure 5) with a small spoon excavator, and a thin layer of luting GIC (Luting and Lining Cement, GC Corporation, Tokyo, Japan) was placed over the root canal filling.
- Gamma loop posts (made of a 0.6 mm stainless steel wire) were placed in 51 and 61. Care was taken to secure the posts with a floss for the fear of accidental aspiration until they were cemented in the canal with the help of luting GIC (Figures 6 and 7). Following this, the GIC and the coronal tooth structure were cleaned with saline, dried, etched (Eco-Etch, Ivoclar Vivadent), washed, dried, and cured after application of the bonding agent (Adper™ Single Bond 2, 3M, ESPE).
- The core and crown buildup was done using free-hand buildup with composite resin (Filtek™ Z350 XT, 3M, ESPE) (Figure 8).
- 52 and 62 were planned for the Ribbond post (Ribbond Inc., Seattle, Washington, USA).
- For this, Vitapex was removed from the coronal portion (3 mm) of the root canal. This length of the root canal was measured using a Williams probe.
- The width of Ribbond was decided based on the root canal space available. A 3 mm wide Ribbond fibre was cut to a length double of this measurement plus an excess of 2-3 mm. Care was taken not to contaminate the Ribbond fibre.
- The root canal was prepared to receive the Ribbond post by etching for 15 seconds (Eco-Etch, Ivoclar Vivadent), washing for 30 seconds, and gently air-drying [6], after which the bonding agent was applied (Adper Single Bond 2, 3M, ESPE) and cured.
- The Ribbond fibre was placed on a paper pad and was coated with a layer of unfilled resin (Clinpro™ Sealant, 3M, ESPE). The excess resin was removed by pressing the Ribbond between the prongs of a pair of tweezers.
- Following this, the length of the fibre was folded over itself and then inserted in the canal so as to maximise the reinforcement of the canal with the fibre (Figure 8).
- Ribbond was stabilised with flowable composite (G-ænial Universal Flo, GC Corporation, Tokyo, Japan), which was then cured. Care was taken to keep 2-3 mm of the fibre above the cementoenamel junction (Figure 9). The protruding ends of the Ribbond strip aided in reinforcing the core buildup that was done to substitute the missing coronal tooth structure. Utmost caution was exercised to make sure that the resin filled the space between the extended Ribbond strips so as not to leave any voids.
- Free-hand composite build up was done to restore the coronal structure.
- Occlusal interferences were checked with an articulating paper, and occlusion was restored. Restorations were finished and polished (Figure 10).
- 54, 55, 64, 65, 74, and 75 were restored with glass ionomer cement (high-strength posterior restorative, GC Corporation, Tokyo, Japan).
- 85 was pulpectomised and restored with a stainless steel crown (Hu-Friedy Pedo Crowns).

## 4. Discussion

In the past, the only treatment option for severely decayed teeth was to extract them and replace them with a prosthesis till the permanent successors erupted. However, with the numerous techniques and materials [2] available now, we are duty-bound to encourage the parents to succumb to extraction only as a last resort while making every effort to salvage these teeth till their natural exfoliation time. The importance of preserving the primary teeth, the role of primary teeth in preventing future malocclusions, and the consequences of premature loss of primary teeth, if explained to the parents well, will lead to more number of primary teeth being restored rather than being extracted. In the present case also, the parents were convinced to save the primary teeth, although they were so critically broken down.

In order to improvise on retention and stress distribution, the post and core were needed as the coronal tooth structure was compromised [7, 8]. The post interconnects the two fragments and minimises stresses on the tooth structure that is being reinforced [9]. The reconstructed crown will be more stable and will be able to endure masticatory forces in function [10]. A diversity of techniques have been used for intracanal reinforcement of anterior teeth, such as metal screw posts, Ni-Cr coil spring posts, short composite posts, biologic posts which are procured from a tooth bank, short wire posts (omega or gamma loop), ready-made glass fibre posts, and polyethylene fibre posts/Ribbond. The coronal tooth structure may be reestablished by direct incremental composite buildup, composite buildup using celluloid strip crowns, indirect composite buildup, and biological shell crowns [2]. In this patient, gamma loop posts were used for the central incisors and Ribbond posts for the lateral incisors, and the coronal structures were replaced with free-hand direct composite buildup.

Prefabricated posts do not follow the discrete contour of the root canal, although they are quick, inexpensive, and easy to use. Even though metal posts can be used for primary teeth, there is an aesthetic concern owing to their colour. Furthermore, these may affect the resorption of the root during the natural exfoliation. Composite posts offer reasonable esthetics; however, the associated and inherent polymerization shrinkage could result in a compromised retention. The accessibility of a tooth bank is a prerequisite for biological posts which are also still a subject to new studies for future conclusions. Wire loops curved in altered shapes, that is, alpha, gamma, and delta, have long been used by many clinicians as posts for primary teeth. Wire curved in the form of alpha is pressure-bonded within the root canals, and this may cause stresses in the dentine. Although with wire curved in the form of gamma, a success rate of 93% has been reported [2].

Composite materials have been reinforced with different fibre types such as carbon fibres, Kevlar fibres, Vectran fibres, glass fibres, and polyethylene fibres. Carbon fibres avert fatigue fracture and fortify composite materials; however, their colour is dark, which makes them objectionable esthetically. Kevlar fibres made of an aromatic polyamide upsurge the impact strength of composites but are unaesthetic and hence have limited use. Vectran fibres are synthetic fibres made of aromatic polyesters. They possess good abrasion resistance and impact strength, but they are expensive and difficult to manipulate [11]. The adhesion of the polyethylene fibre post to the composite resin matrix is better when compared to the adhesion of the glass fibre post to composite resin. Ribbond fibre posts offer good impact strength to composite resin used for coronal reconstruction. This is because of their modulus of elasticity and flexural strength being close to dentine [2]. In Ribbond, the fibres are not arranged longitudinally and are instead woven in alternating patterns. This arrangement results in improved distribution of the internal tension lines and thus provides fracture resistance [12].

The advantage of using the reinforced composite material as an intracanal post includes resin composite crown reinforcement, translucency, and relative ease of manipulation [11]. The resin adapts to the intimate shape of the canal space ensuring that there would be negligible, if any, voids. It bonds to the resin which is used for building the core and the crown, and hence, it results in the creation of a single block of the post, core, and crown. Thus, there is excellent resistance against debonding of the entire unit and also favourable occlusal force transmission. Another advantage of using Ribbond as a post is that there is no metal which needs to be masked while building the core and crown with composite. After the Ribbond (although opaque in nature) is completely enclosed in the composite, it does not adversely affect the colour of the core or the crown [13]. According to Memarpour et al. [14], polyethylene posts associated with extensive composite restoration show excellent clinical performance.

## 5. Conclusion

The importance of retaining the primary anterior teeth till their natural exfoliation time cannot be overemphasized. It plays a pivotal role in maintaining esthetics, development of speech, and building up of a confident individual.
